# Artificial intelligence evaluation of electrocardiographic characteristics and interval changes in transgender patients on gender-affirming hormone therapy

**DOI:** 10.1093/ehjdh/ztae076

**Published:** 2024-10-15

**Authors:** Fadi W Adel, Philip Sang, Connor Walsh, Arvind Maheshwari, Paige Cummings, Zachi Attia, Kathryn Mangold, Caroline Davidge-Pitts, Francisco Lopez-Jimenez, Paul Friedman, Peter A Noseworthy, Rekha Mankad

**Affiliations:** Department of Cardiovascular Medicine, Mayo Clinic, 200 1st St SW, Rochester, MN 55905, USA; Department of Internal Medicine, Mayo Clinic, 200 1st St SW, Rochester, MN 55905, USA; Department of Internal Medicine, University of Washington, 2505 2nd Ave, Seattle, WA 98121, USA; Advocate Medical Group, 27750 West Highway 22 Suite 110, Barrington, IL 60010, USA; Mayo Clinic Alix School of Medicine, Mayo Clinic, 200 1st St SW, Rochester, MN 55905, USA; Department of Cardiovascular Medicine, Mayo Clinic, 200 1st St SW, Rochester, MN 55905, USA; Department of Cardiovascular Medicine, Mayo Clinic, 200 1st St SW, Rochester, MN 55905, USA; Division of Endocrinology, Diabetes, and Nutrition, Department of Medicine, Mayo Clinic, 200 1st St SW, Rochester, MN 55905, USA; Department of Cardiovascular Medicine, Mayo Clinic, 200 1st St SW, Rochester, MN 55905, USA; Department of Cardiovascular Medicine, Mayo Clinic, 200 1st St SW, Rochester, MN 55905, USA; Department of Cardiovascular Medicine, Mayo Clinic, 200 1st St SW, Rochester, MN 55905, USA; Department of Cardiovascular Medicine, Mayo Clinic, 200 1st St SW, Rochester, MN 55905, USA

**Keywords:** Transgender individuals, Artificial intelligence, Electrocardiogram, LGBTQIA+ Health, Gender-affirming hormone therapy

## Abstract

**Aims:**

Gender-affirming hormone therapy (GAHT) is used by some transgender individuals (TG), who comprise 1.4% of US population. However, the effects of GAHT on electrocardiogram (ECG) remain unknown. The objective is to assess the effects of GAHT on ECG changes in TG.

**Methods and results:**

Twelve-lead ECGs of TG on GAHT at the Mayo Clinic were inspected using a validated artificial intelligence (AI) algorithm. The algorithm assigns a patient’s ECG male pattern probability on a scale of 0 (female) to 1 (male). In the primary analysis, done separately for transgender women (TGW) and transgender men (TGM), 12-lead ECGs were used to estimate the male pattern probability before and after GAHT. In a subanalysis, only patients with both pre- and post-GAHT EGCs were included. Further, the autopopulated PR, QRS, and QTc intervals were compared before and after GAHT. Among TGW (*n* = 86), the probability (mean ± SD) of an ECG male pattern was 0.84 ± 0.25 in the pre-GAHT group, and it was lowered to 0.59 ± 0.36 in the post-GAHT group (*n* = 173, *P* < 7.8 × 10^−10^). Conversely, among TGM, male pattern probability was 0.16 ± 0.28 (*n* = 47) in the pre-GAHT group, and it was higher at 0.41 ± 0.38 in the post-GAHT group (*n* = 53, *P* < 2.4×10^−4^). The trend persisted in the subanalysis. Furthermore, both the PR (*P* = 5.68 × 10^−4^) and QTc intervals (*P* = 6.65×10^−6^) prolonged among TGW. Among TGM, the QTc interval shortened (*P* = 4.8 × 10^−2^).

**Conclusion:**

Among TG, GAHT is associated with ECG changes trending towards gender congruence, as determined by the AI algorithm and ECG intervals. Prospective studies are warranted to understand GAHT effects on cardiac structure and function.

## Graphical Abstract

**Graphical Abstract:**
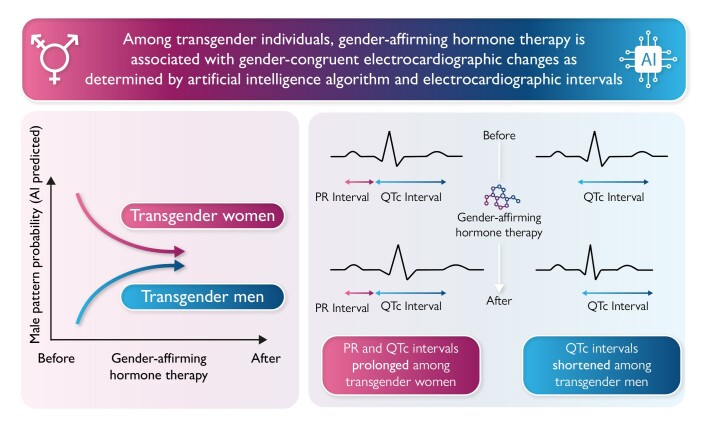

## Introduction

Transgender individuals (TG), whose sex recorded at birth is incongruent with their gender identity, constitute ∼1.6 million individuals in the USA, with anticipated increased recognition and visibility of this population.^1^ Transgender women (TGW) are recorded male at birth and identify as female/feminine. Transgender men (TGM) are recorded female at birth and identify as male/masculine.^2,3^

Gender-affirming hormone therapy (GAHT) is sought by a considerable number of TG, and it can have important cardiovascular implications, with previous reviews suggesting potentially increased rates of myocardial infarction, stroke, and venous thrombo-embolic disease.^4–6^ Therefore, it becomes increasingly important to characterize the effects of GAHT on cardiovascular parameters among TG accurately.

Binary cardiac structural and electrocardiogram (ECG)-based differences have long been assumed in the cisgender population, including longer QTc and shorter PR and QRS intervals among cisgender women (CGW).^7–9^ These changes have been proposed to be due to the effects of sex hormones on cardiac ion channels and myocardial tissue overall. For instance, testosterone inhibits the inward L-type calcium current (*I*_CaL_) during the early repolarization phase^10^ and may enhance the outward potassium currents (*I*_kr_ and *I*_ks_) in the late repolarization phase, potentially explaining the shorter QTc intervals among cisgender men.^11^

Further, a cutting-edge artificial intelligence (AI)-based algorithm developed at the Mayo Clinic has been shown to predict a cisgender patient's sex with 90.4% accuracy.^12^ In addition, the same algorithm has been adapted to predict atrial fibrillation^13^ and cardiac amyloidosis.^14^ However, our knowledge pertaining to cardiovascular changes among GAHT-using TG remains sparse.^3^ Indeed, without a clear understanding of the cardiovascular profile of TG, clinicians have an unclear vision of how to treat them. For instance, among mostly GAHT-using TGW, the average functional aerobic capacity was fair compared with cisgender men and average compared with CGW.^15^ In that same cohort, the stress echocardiography profile of TGW was unique compared with their cisgender counterparts.^16^ Nevertheless, it remains largely unclear whether the use of GAHT in TG is associated with gender-congruent electrocardiographic changes, either through ECG intervals or AI predictions.

In this study, we focused on exploring the GAHT-associated electrocardiographic changes using a cutting-edge AI-based algorithm, as well as inspecting the ECG interval changes. By leveraging this capability, we examined whether the algorithm detected any changes to the electrical patterns of the hearts of TG as determined by ECG in this proof-of-concept project.

## Methods

### Study design

This was a descriptive study comparing the AI-predicted ECG male patterns in TG before and after GAHT initiation in a single tertiary care centre. The study was approved by institutional board review at the Mayo Clinic in Rochester, MN, USA.

### Definitions

To define the gender identity of the study sample, we employed the gender-affirming terminology recommended by the American Heart Association.^3^

Cisgender men (CGM) are persons whose gender male/masculine identity is congruent with the male sex recorded at birth. Cisgender women are individuals whose female/feminine gender identity is congruent with the female sex recorded at birth.^2,3^

The gender identity of TG is incongruent with their sex recorded at birth. Transgender women (TGW) are individuals whose gender identity is female/feminine and whose sex recoded at birth is male. Transgender women may use feminizing GAHT.^2,3^

Transgender men (TGM) are individuals whose gender identity is male/masculine and whose sex recorded at birth is female/feminine, and they may use masculinizing GAHT.^2,3^

### Population and setting

The Transgender and Intersex Specialty Care Clinic (TISCC) is a multidisciplinary clinic that offers gender-affirming medical, psychosocial, and surgical expertise to TG at the Mayo Clinic in Rochester, MN, USA.^17^ The TISCC maintains a database of patients who received care at the clinic and consented to have their charts used for research. We extracted ECG data from the Mayo Clinic electronic medical record, focusing on charts of self-identified TGM and TGW over the age of 18. Only GAHT-utilizing, self-identified TGW and TGM were included in this study. Individuals identifying as gender non-binary were not included in this study. Patients with cardiovascular comorbidities were not excluded from the study. The ECGs were obtained for clinical purposes, and the specific indications for obtaining those ECGs were not extracted. Pertinent data, including demographics, comorbidities (as listed in the electronic medical record), cardioactive medications, GAHT regimens, and dates of GAHT initiation, were collected.

Patients were then divided into those who only have ECGs before GAHT initiation, those who only have ECG after GAHT initiation, and those who have ECGs both before and after GAHT initiation.

Electrocardiograms were then inspected using the AI algorithm. In the main analysis, all 12-lead ECGs before GAHT were used to estimate the pre-GAHT male pattern probability, while all 12-lead ECGs after GAHT initiation were used to estimate the male pattern probability. This analysis was performed separately among TGM and TGW.

In a similar analysis of a subset of patients, only TG who had 12-lead ECGs recorded in the electronic system prior to and following the initiation of gender-affirming hormone therapy (GAHT) were included.

### Artificial intelligence algorithm

The AI-ECG sex model was developed at Mayo Clinic as previously described in Attia *et al*.^12^ Briefly, the model used raw 10s 12-lead ECGs as input and extracted features through sequential convolutional blocks to yield probabilities of male and female sexes. The model development patient cohort consisted of 774 783 patients (52% male) with 275 056 of these belonging to the holdout testing set. The testing set’s male and female probabilities classified sex with area under the curve of 0.97 with an overall accuracy of 90.4% (95% confidence interval: 90.3–90.5%).

### Electrocardiogram intervals

Electrocardiographic intervals were automatically autopopulated from the ECG data collected for each individual and were not manually measured by the authors. The units of measurement were milliseconds (ms). The intervals were treated as a continuous variable.

### Statistical analysis

To determine whether the mean male pattern probability differed before and after GAHT for all 12-lead ECGs, male probabilities from the first ECG on record per patient before GAHT were compared with male probabilities from the first ECG per patient after GAHT. Independent sample *t*-tests without equal variances were used to test for significant differences in the mean male pattern probability for the overall cohort. For patients with ECGs recorded both before and after GAHT at Mayo, the differences in the mean male pattern probability were determined using the dependent sample (paired) *t*-tests. Independent and dependent *t*-tests were run in Scipy 1.7.1. Given the multiple comparisons, we adjusted the significance level using the Benjamini–Hochberg adjustment method.

## Results

The baseline characteristics of both TGW and TGM, including their comorbidities, cardiovascular medications, and GAHT regimens, are listed in *Table 1*. The mean age among TGW (*n* = 208) was 45.5 ± 17.1 years, and it was 31.3 ± 8.6 years among TGM (*n* = 80). It is noteworthy that no comparisons were made between the two cohorts since it was not the objective of this study.

**Table 1:** Patient characteristics

	TGW (*n* = 208)	TGM (*n* = 80)
Age (years), mean ± SD	45.5 ± 17.1	31.3 ± 11.9
BMI, mean ± SD	30.0 ± 8.9	31.1 ± 8.6
Comorbidities		
Hypertension, *n* (%)	51 (24.5)	13 (16.3)
Hyperlipidaemia, *n* (%)	56 (26.9)	15 (18.8)
Diabetes, *n* (%)	24 (11.5)	5 (6.3)
Coronary artery disease, *n* (%)	16 (7.7)	1 (1.3)
History of myocardial infarction, *n* (%)	4(1.9)	0
Heart failure (diastolic or systolic), *n* (%)	2(1.0)	0
Atrial fibrillation/flutter, *n* (%)	4(1.9)	0
CKD ≥ stage 3, *n* (%)	13 (6.3)	2 (2.5)
Thyroid disease, *n* (%)	12 (5.8)	9 (11.3)
Peripheral arterial disease, *n* (%)	1 (0.5)	0
Medications		
Statin, *n* (%)	59 (28.4)	7 (8.8)
Diuretic, *n* (%)	19 (9.1)	3 (3.8)
ACEi, *n* (%)	18 (8.7)	2 (2.5)
ARB, *n* (%)	8 (3.8)	3 (3.8)
Beta-blocker, *n* (%)	19 (9.1)	11 (13.8)
Calcium channel blocker, *n* (%)	13 (6.3)	7 (8.8)
Aspirin, *n* (%)	31 (14.9)	4 (5.0)
P2Y12 inhibitor, *n* (%)	3 (1.4)	0
Anticoagulant, *n* (%)	7 (3.4)	0
Long-acting nitrate, *n* (%)	7 (3.4)	1 (1.3)
GAHT		
Spironolactone, *n* (%)	95 (45.7)	1 (1.3)
Oestrogen therapy		
Injection, *n* (%)	77 (37.0)	
Oral, *n* (%)	81 (38.9)	
Transdermal, *n* (%)	48 (23.1)	
Progesterone, *n* (%)	81 (38.9)	
5-ARI, *n* (%)	45 (21.6)	
Testosterone therapy		
Subcutaneous, testosterone cypionate, *n* (%)		36 (46.7)
Subcutaneous, testosterone enanthate, *n* (%)		8 (10.4)
Intramuscular, testosterone cypionate, *n* (%)		29 (37.7)
Intramuscular, testosterone enanthate, *n* (%)		2 (2.6)
** **Gel, *n* (%)		2 (2.6)

BMI, body mass index; CKD, chronic kidney disease; ACEi, angiotensin-converting enzyme inhibitor; ARB, angiotensin-receptor blocker; GAHT, gender-affirming hormone therapy; 5-ARI, 5α-reductase inhibitor.

Among TGW, the most prevalent cardiovascular comorbidities included hyperlipidaemia (26.9%), hypertension (24.5%), coronary artery disease (16%), and diabetes (11.5%). Gender-affirming hormone therapy included different oestrogen formulations, spironolactone, progesterone, and 5α-reductase inhibitor (*Table 1*).

Among TGM, the comorbidities followed a similar pattern, with hyperlipidaemia (18.8%) being most common, followed by hypertension (16.3%), diabetes (6.3%), and coronary artery disease (1.3%). The majority of patients were on testosterone (*Table 1*) therapy.

### Probability of male pattern in overall cohort

**Figure 1:**
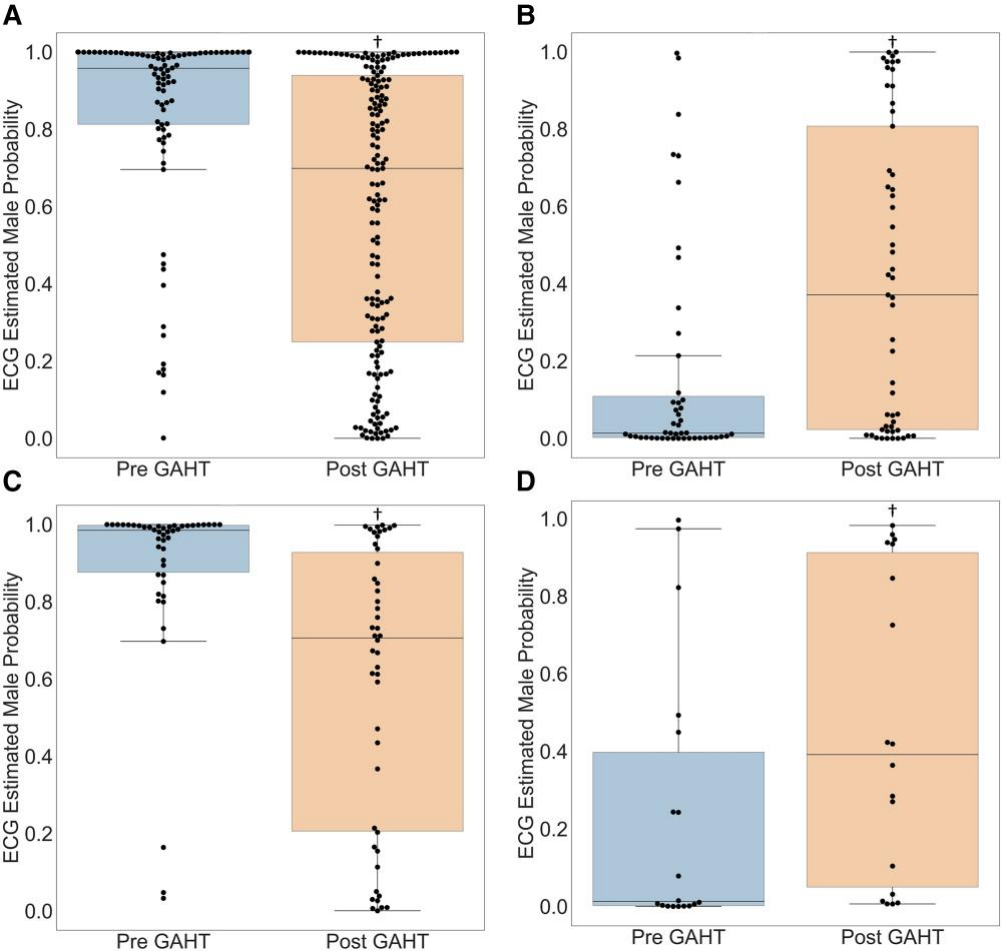
Electrocardiogram artificial intelligence algorithm–estimated probability of male pattern. The artificial intelligence algorithm predicts the probability of a male pattern, ranging from 0 to 1, with 0 being more consistent with a cisgender women electrocardiogram pattern and 1 being more consistent with a cisgender men one. In the overall cohort of transgender women (*A*), the male pattern probability (mean ± SD) was 0.84 ± 0.25 before feminizing gender-affirming hormone therapy (left boxplot, *n* = 86), and it decreased to 0.59 ± 0.36 with gender-affirming hormone therapy (right boxplot, *n* = 173), shifting away from male electrocardiogram patterns (*P* < 7.8 × 10^−10^). Conversely, in the cohort of transgender men (*B*), the probability of a male pattern increased from 0.16 ± 0.28 (left boxplot, *n* = 47) to 0.41 ± 0.38 (right boxplot, *n* = 53) following masculinizing gender-affirming hormone therapy, shifting towards a male pattern (*P* < 2.4×10^−4^). Using only the electrocardiograms of patients who have both pre– and post–gender-affirming hormone therapy electrocardiograms in our records, we performed a similar analysis. Similar to the overall cohort, among transgender women (*n* = 46, *C*), the probability of a male electrocardiogram pattern decreased from 0.88 ± 0.23 (left boxplot) to 0.59 ± 0.36 (right boxplots, *P* < 2.4 × 10^−6^). Similarly, in an analogous pattern to the overall cohort, that probability increased from 0.24 ± 0.35 (left boxplots) to 0.45 ± 0.39 (right boxplots, *P* < 0.023) among transgender men (*n* = 18, *D*). ^†^*P*-value < 0.048.

In the cohort of TGW, the probability of a male pattern (mean ± SD) was 0.84 ± 0.25 in the group with pre-GAHT data (*n* = 86), while it was lower at 0.59 ± 0.36 (*n* = 173) in the group with post-GAHT data (*P* < 7.8 × 10^−10^) (*Figure 1A*). On the other hand, in the cohort of TGM, the male pattern probability among those with pre-GAHT data was 0.16 ± 0.28 (*n* = 47), and it was higher at 0.41 ± 0.38 (*n* = 53) among those with post-GAHT data (*P* < 2.4×10^−4^) (*Figure 1B*).

### Probability of male pattern in pre– and post–gender-affirming hormone therapy electrocardiogram cohort only

The aforementioned trend was sustained when the analysis included participants with available ECGs before and after GAHT. Among TGW (*n* = 46), the average probability of a male pattern decreased from 0.88 ± 0.23 to 0.59 ± 0.36 (*P* < 2.4 × 10^−6^) (*Figure 1C*), while it rose from 0.24 ± 0.35 to 0.45 ± 0.39 (*P* < 0.023) among TGM (*n* = 18) (*Figure 1D*).

### Gender-affirming hormone therapy–associated electrocardiogram interval changes in overall cohort

**Figure 2:**
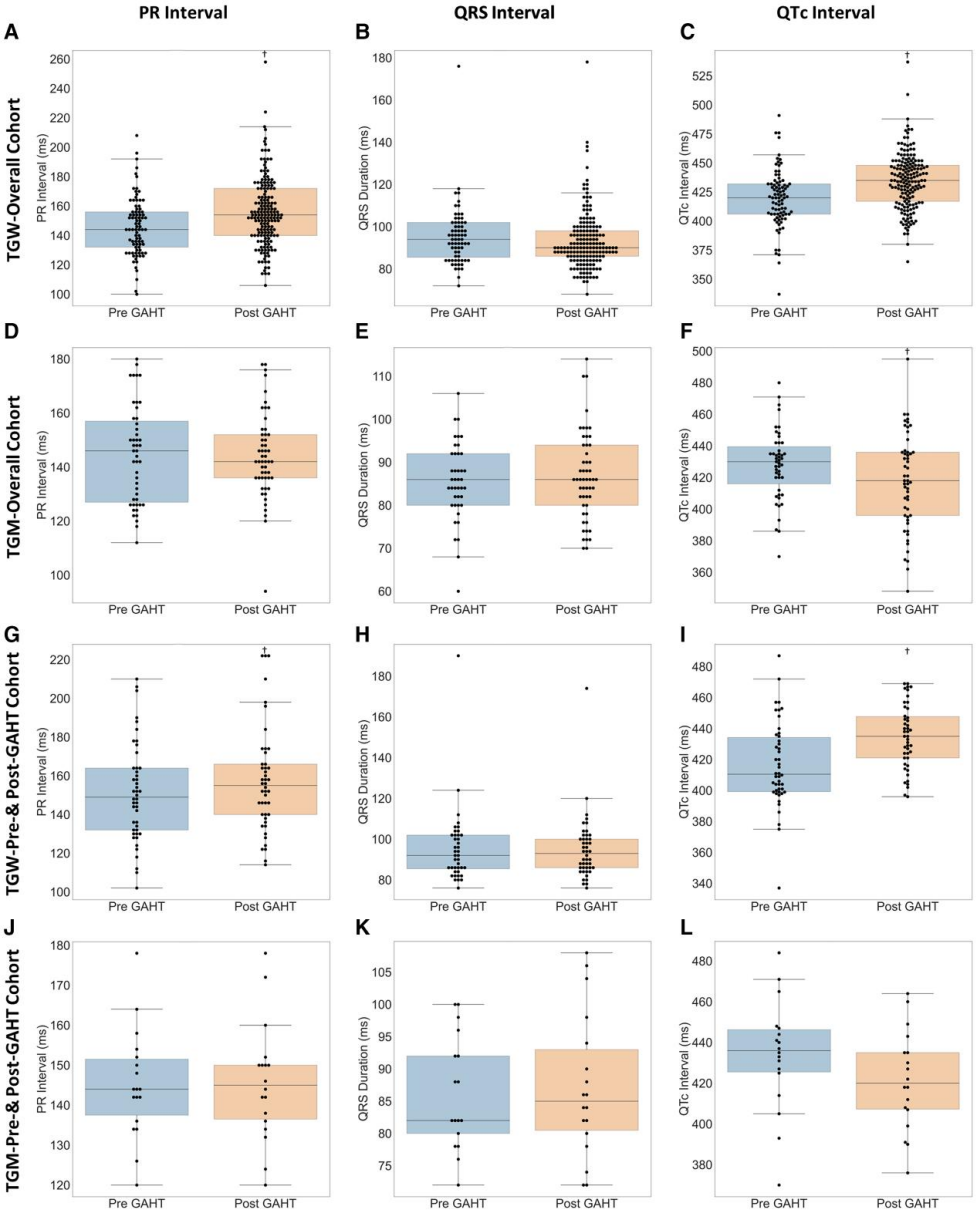
Electrocardiogram interval change with gender-affirming hormone therapy. Among transgender women in the overall cohort, gender-affirming hormone therapy was associated with prolongation of both the PR (*A*) and the QTc (*C*) intervals without a significant change in the QRS interval (*B*). Among transgender men in the overall cohort, there were no changes in the PR (*D*) or QRS (*E*) intervals, but the QTc interval decreased (*F*). In the subanalysis including only patients with both pre– and post–gender-affirming hormone therapy ECGs, a similar trend was observed, with PR (*G*) and QTc (*I*) interval prolongation without a change in QRS (*H*) duration among transgender women. While neither the PR (*J*) nor the QRS (*K*) intervals changed in transgender men in the subanalysis, there was a trend towards QTc interval shortening (*L*). ^†^*P*-value < 0.048.

Given the known, sex-based electrocardiographic differences within the cisgender population, we investigated whether GAHT was associated with any PR, QRS, or QTc interval changes. In the primary analysis, the ECG of TGW revealed prolongation of both the PR interval (pre-GAHT: 146.20 ± 19.97 ms to post-GAHT: 155.97 ± 23.27 ms, *P* = 5.68 × 10^−4^, *Figure 2A*) and the QTc interval (pre-GAHT: 419.53 ± 24.99 ms to post-GAHT: 434.72 ± 24.17 ms, *P* = 6.65×10^−6^, *Figure 2C*), without a significant change in QRS duration (*Figure 2B*).

Among TGM, there was shortening of the QTc interval (pre-GAHT: 427.87 ± 22.17 ms to post-GAHT: 417.36 ± 30.13, *P* = 4.8 × 10^−2^, *Figure 2F*) without significant changes in the PR (*Figure 2D*) or QRS (*Figure 2E*) intervals.

### Gender-affirming hormone therapy–associated electrocardiogram interval changes in pre– and post–gender-affirming hormone therapy electrocardiogram cohort only

Similarly, the ECG of TGW demonstrated an increase in the PR (pre-GAHT: 151.17 ± 25.39 ms to post-GAHT: 157.43 ± 27.01 ms, *P* = 9.19 × 10^−3^, *Figure 2G*) and the QTc (pre-GAHT: 416.63 ± 27.47 ms to post-GAHT: 434.63 ± 20.66 ms, *P* = 4.29 ×10^−5^, *Figure 2I*) intervals, without any changes in the QRS duration (*Figure 2H*).

Among TGM, there was a trend towards shortening of the QTc interval (pre-GAHT: 433.89 ± 27.17 ms to post-GAHT: 421.33 ± 24.23, *P* = 0.08, *Figure 2L*), without any significant changes in the PR (*Figure 2J*) or QRS (*Figure 2K*) intervals.

## Discussion

On average, TG are at an increased risk of worse cardiovascular outcomes than their cisgender counterparts.^3^ Yet, knowledge about their overall cardiovascular health and cardiovascular risk stratification remains in dearth. For the first time, we demonstrated in our study that the AI-predicted male pattern probability on ECG among TGW decreases with the use of feminizing GAHT, and it increases among TGM with the use of masculinizing GAHT. Further, GAHT is associated with gender-congruent changes in QTc intervals, as well as PR prolongation among TGW. These preliminary findings point towards a number of potential implications, and they emphasize the need for further robust research in the field of transgender health.

Among cisgender individuals, binary sex-based ECG differences have long been recognized, such as shorter QTc intervals among CGM^7,9,18,19^ and shorter QRS and PR intervals among CGW.^9,19^ In a recent small study, GAHT was indeed associated with ECG changes that were congruent with the affirmed gender of TG.^20^ In our study, we employed a novel ECG AI algorithm that recognizes binary sex-based patterns that are not distinguishable to the clinician’s naked eye^12^ to the ECGs of TG. Additionally, we showed that the QTc interval evolves congruently with GAHT, prolonging in TGW and shortening in TGM while the PR interval prolongs in TGW. Collectively, our findings reveal changes in the electrical footprint of the heart as detected by the ECG, demonstrated by the ECG interval changes and the ‘unseen’ AI-detected changes in male pattern probability.

The change in the electrocardiographic footprint of the heart may imply a change on a cellular level within the myocardium. Sex hormones are known to induce effects on the myocardium. For instance, testosterone has been shown to be associated with cardiomyocyte hypertrophy in both sexes and hyperplasia in mammals,^21^ and it is associated with QTc shortening via impacting potassium channel densities.^22^ On the other hand, oestrogen has been associated with antihypertrophic^23^ and anti-apoptotic effects^24^ on the myocardium. Indeed, one might hypothesize that the AI-predicted probability is a reflection of the combination of the chromosomal and the hormonal milieu. In other words, in CGW, the ECG signature can reflect the myocardium of an XX individual in a milieu of largely oestrogen and progesterone. In TGW, the ECG signature may reflect the XY myocardium in a milieu of largely oestrogen and progesterone. Whether the latter would result in a completely gender-congruent or simply trend it to a non-binary status remains to be determined via longer, prospective studies. The fact that ECG sex pattern evolves with GAHT implies that structural myocardial changes could be occurring concomitantly; these suggestions indicate that perhaps TG have a unique cardiovascular profile different from their cisgender counterparts and that clinicians should be careful when using tools and parameters validated using cisgender individuals.

Indeed, this is further supported by the evolution of the QTc interval in TGM and TGW, and the PR interval prolongation in TGW. After puberty, the QT interval becomes longer in CGW compared with CGM,^7,25^ and these changes become attenuated with age.^26^ Additionally, among heart transplant recipients whose sex is different from that of the donor, the QTc evolves to conform to the sex of the recipient,^27^ further supporting the role the hormonal milieu plays in myocardial changes. Consistent with these findings are our observations of QTc prolongation in TGW and QTc shortening in TGM.

On average, the PR interval is shorter in women compared with men in the cisgender population.^19,28^ The PR interval denotes intra-atrial depolarization and atrioventricular nodal conduction speed, and it is affected by the atrial structure and the autonomic and hormonal milieus.^28^ In TGW, the fact that the PR interval prolongs is certainly interesting and it supports the notion that perhaps TGW have a distinct electrocardiographic footprint. Indeed, in a recent study, TGW exhibited a distinct stress echocardiographic profile compared with their cisgender counterparts.^16^

Certain cardiovascular tools utilize specific reference ranges that are sex-based, such as functional aerobic capacity (FAC) and atherosclerotic cardiovascular risk algorithms. In addition, several echocardiographic parameters, such as left ventricular mass and chamber dimensions, are sex-based.^29^ Without validated models based on TG, clinicians revert to using tools that were validated on cisgender individuals. Using those references may over- or underestimate the results obtained in TG. For instance, in a cohort of TGW, the mean FAC was fair when compared with CGM and average when compared with CGW.^15^ Similarly, using the pooled-cohort equation to estimate the 10-year atherosclerotic risk, atherosclerotic risk estimates were significantly different when the authors used male vs. female in the calculator.^15^

The inconsistent performance of standard risk stratification schemes may add to the quality gap in healthcare among TG. Indeed, TG face considerable health disparities across many cardiovascular risk factors at baseline.^30^ Further, feminizing GAHT has been associated with increased risk of venous thrombo-embolism^31^ and masculinizing GAHT has been associated with increased myocardial infarctions^32^ and reduced endothelial function.^33^ Therefore, establishing and using TG-specific accurate risk estimators and reference ranges becomes very important.

Our study emphasizes the potential of AI-driven algorithms to identify GAHT-associated cardiovascular changes that may not be evident through traditional ECG interpretation. While serving as a proof of concept, this study underscores the need for future studies to validate AI-driven tools in larger cohorts. Eventually, these innovations could lead to TG-specific reference ranges and improved cardiovascular risk estimation tools, ultimately enhancing clinical outcomes for transgender patients. Of note, the ECGs were obtained for clinical purposes, and the specific indications for obtaining these ECGs were not extracted. While assessing electrocardiographic activity in a real-world setting offers valuable insights, it complicates the precise isolation of hormonal effects. Future research could benefit from focusing on ECGs from a subgroup of patients without cardiovascular disease to better delineate these hormonal influences.

Collectively, our results reveal that GAHT is associated with cardiac electrical pattern changes in TG, emphasizing the need for further robust research into cardiovascular parameters validated among TG.

### Limitations

This was a retrospective study in a single tertiary care centre, which could introduce selection bias to the sample. Moreover, there is a potential selection bias regarding individuals who receive ECGs, as their indications are unknown, potentially differing from those who do not receive ECGs. Furthermore, hormonal levels and GAHT duration were not assessed among the participants, limiting the ability to investigate the association between ECG changes and these factors. Due to some constraints, namely data availability, sample size limitations, risk of encountering false positive conclusions, and heterogeneity in treatment regimens, we were unable to determine the specific effects of each subtype of GAHT or dosing of GAHT on ECG changes. Additionally, no cisgender comparators were included in this study to definitively discern whether those changes are due to GAHT and not due to age. Moreover, the AI algorithm's performance among TGM and TGW pre-GAHT is slightly different from its performance among cisgender individuals, which may be related to the training population being primarily cisgender. The rationale for this difference is unclear. Prospective studies comparing the male pattern probability between GAHT-naive TG and age-matched cisgender counterparts are needed to better understand these observations.

## Conclusion

Electrocardiographic changes, primarily trending towards gender congruence, develop among TG individuals using GAHT, as determined by AI and ECG interval changes. Future, prospective, multicentre studies are warranted to further inspect the effects of GAHT on myocardial structure and function.

## Data Availability

The data supporting the findings of this study are available from the corresponding author, R.M., upon reasonable request.
